# Interfacial Friction Anisotropy in Few-Layer Van der Waals Crystals

**DOI:** 10.3390/ma14164717

**Published:** 2021-08-20

**Authors:** Kaibo Wang, Hao Li, Yufeng Guo

**Affiliations:** State Key Laboratory of Mechanics and Control of Mechanical Structures and MOE Key Laboratory for Intelligent Nano Materials and Devices, College of Aerospace Engineering, Nanjing University of Aeronautics and Astronautics, Nanjing 210016, China; wangkb@nuaa.edu.cn (K.W.); zhouxue@nuaa.edu.cn (H.L.)

**Keywords:** two-dimensional crystals, friction anisotropy, pressure, thickness, first-principles calculations

## Abstract

Friction anisotropy is one of the important friction behaviors for two-dimensional (2D) van der Waals (vdW) crystals. The effects of normal pressure and thickness on the interfacial friction anisotropy in few-layer graphene, *h*-BN, and MoSe_2_ under constant normal force mode have been extensively investigated by first-principle calculations. The increase of normal pressure and layer number enhances the interfacial friction anisotropy for graphene and *h*-BN but weakens that for MoSe_2_. Such significant deviations in the interfacial friction anisotropy of few-layer graphene, *h*-BN and MoSe_2_ can be mainly attributed to the opposite contributions of electron kinetic energies and electrostatic energies to the sliding energy barriers and different interlayer charge exchanges. Our results deepen the understanding of the influence of external loading and thickness on the friction properties of 2D vdW crystals.

## 1. Introduction

Layered van der Waals (vdW) materials such as graphite, boron nitride, and transition metal dichalcogenides (TMDs) which layers bind with weak interlayer vdW interactions, have been widely used as solid lubricants in engineering technology to reduce friction and wear. When size goes down to nanoscale, two-dimensional (2D) vdW crystals exhibit exceptional and excellent friction properties and have attracted numerous scientific interests [1,2,3,4,5,6,7,8,9,10,11,12,13,14,15,16,17,18,19,20,21,22,23,24,25,26,27,28,29,30,31]. Lee et al. [1] used atomic force microscopy (AFM) technique to characterize the microscopic friction characteristics of monolayer and multilayer graphene, MoS_2_, *h*-BN, and NbSe_2_ that were mechanically peeled off from weakly adherent SiO_2_ substrates, and reveal that the friction force decreases with the increase of the number of layers. Zhang et al. studied the friction behavior of a graphene flake sliding on a supported graphene substrate and showed that the friction force increases exponentially with the decreasing stiffness [2,3,4,5]. Guo et al. [6] find that the interlayer friction force of graphene sheets at the commensurate state increases sharply with the decrease of the interlayer distance while the incommensurate contact maintains an ultra-low friction state. By using a pressurized bubble loading device, Wang et al. [7] found the existence of ultra-low friction characteristics in the incommensurate graphene/graphene interface at the microscopic scale. Negative friction coefficients are found at the interfaces of layered graphene-hexagonal boron nitride (*h*-BN) heterojunctions due to load-induced suppression of out-of-plane distortions [8], and the interfaces between graphene sheets and the tip of atomic force microscope tip [9,10]. The novel friction properties emerging in 2D vdW crystals make them possessing a promising future in the application of functional devices and nanoelectromechanical systems.

Due to surface morphology, crystal lattice, or structural deformation, the friction along various directions is usually different. Friction anisotropy is one of the important friction behaviors and has been extensively studied for solid interfaces and surfaces by theoretical and experimental methods. For 2D vdW crystals, the friction anisotropy also becomes obvious and remarkable [32,33,34,35,36,37,38,39,40,41,42,43,44,45,46]. Nevertheless, most previous first-principles studies on the interlayer friction for two 2D vdW crystals were conducted with constant interlayer distance mode rather than constant normal force mode. Empirical potential based molecular dynamics simulations are hard to accurately describe interlayer coulomb interaction and charge exchange under compression. It is necessary to use more accurate methods to investigate the interfacial friction behaviors of 2D vdW crystals in the presence of ideal constant normal force. On the other hand, mechanical loading is an effective way to modify the physical and chemical properties of low-dimensional materials. Monolayer and few-layer vdW crystals usually possess different friction behaviors. However, the effects of normal pressure and thickness on the interlayer friction anisotropy of few-layer 2D vdW crystals have been seldom studied and remains elusive.

In this work, the interfacial friction properties of few-layer graphene, *h*-BN, and MoSe_2_ under constant normal force mode have been extensively studied by using first-principles calculations. It is found that the deviations in the energy barriers between the interlayer maximum energy sliding paths and minimum energy sliding paths increase for graphene and *h*-BN but decrease for MoSe_2_ with the applied normal pressure increases. The friction anisotropy in graphene and *h*-BN is therefore enhanced but reduced in MoSe_2_ by the normal pressure. Moreover, the increase of layer number increases the friction anisotropy in graphene and *h*-BN and decreases that in MoSe_2_. The significant difference in the influence of normal pressure and layer number on the friction anisotropy among these three kinds of 2D vdW crystals can be attributed to different interlayer charge exchanges and the opposite changes in electron kinetic energies and electrostatic energies. Our results deepen the understanding of the effects of normal pressure and thickness on the friction behaviors of 2D vdW crystals.

## 2. Methods

**Figure 1:**
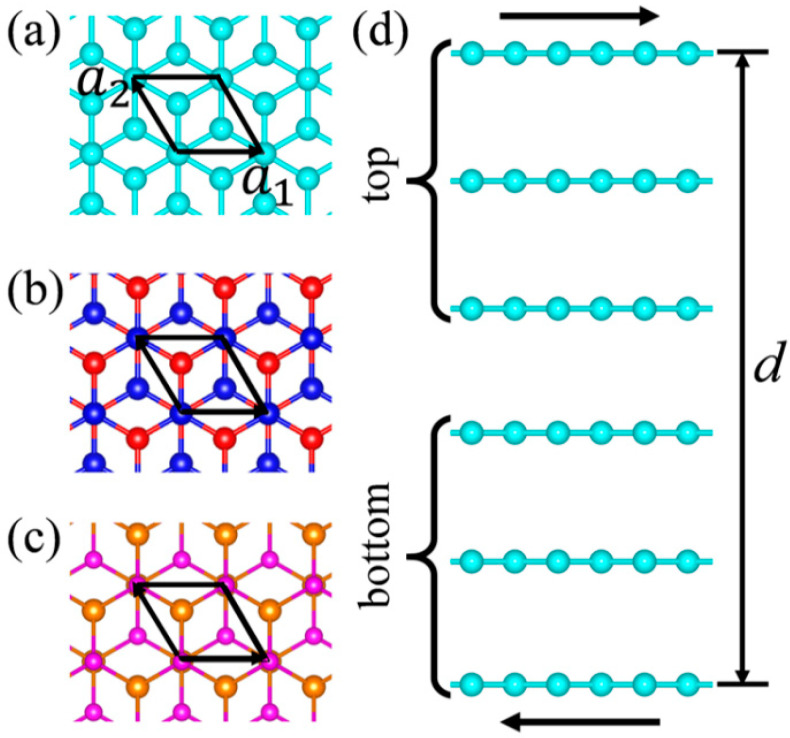
Top views of atomic structures of: (**a**) graphene, (**b**) *h*-BN, and (**c**) MoSe_2_ with AB stacking. (**d**) Side view of a 6*L*-layer graphene. The cyan, blue, red, gold, and pink balls are C, N, B, Mo, and Se atoms, respectively. Here *a*_1_ and *a*_2_ are two lattice vectors of the unit cell, respectively, and *d* is the interlayer distance between the top and bottom layers.

In our model, we established 2 to 8-layer graphene, *h*-BN and 2H MoSe_2_ with interlayer AB stacking in the rhombus unit cells, where graphene, *h*-BN and MoSe_2_ monolayers consist of two C atoms, 1 B, and 1 N atom, and 1 Mo and 2 Se atoms, respectively. Figure 1 shows the atom structures of six-layer graphene, *h*-BN and MoSe_2_. In the unit cells, a vacuum region larger than 15 Å is in the direction perpendicular to the atomic plane. All computations were performed within the framework of density-functional theory (DFT) as implemented in the FHI-aims code with “tight” computational settings [47] in which the Perdew–Burke–Ernzerhof (PBE) [48] was employed. The influence of vdW interactions was considered by using a many body dispersion (MBD) vdW model [49,50]. A *k* point grid of 15 × 15 × 1 was used throughout the work. First the whole systems were relaxed by PBE + MBD until the force on each atom was less than 0.01 eV/Å. The optimized lattice constants of graphene, *h*-BN and MoSe_2_ unit cells are 2.467, 2.507, and 3.28 Å, respectively.

**Figure 2:**
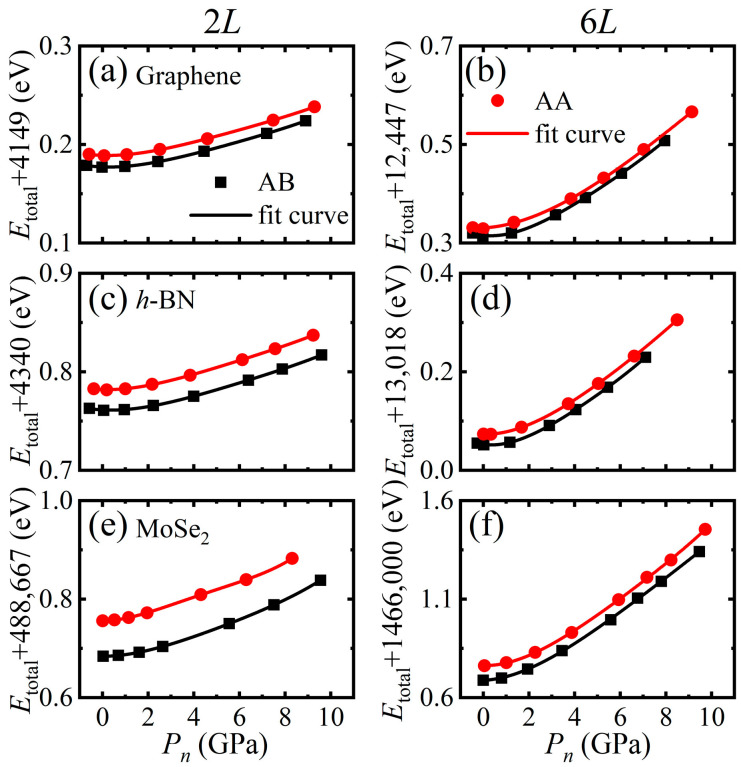
The total energies (in unit of eV) and corresponding fitting curves with normal pressure for 2*L*- and 6*L*-layer: (**a**,**b**) graphene, (**c**,**d**) *h*-BN, and (**e**,**f**) MoSe_2_ with AB and AA stacking.

To simulate interlayer sliding, the whole *a*_1_–*a*_2_ planes of the unit cells were equally divided into 81 positions where the nearest translational positions were separated by 0.274, 0.279, and 0.364 Å for graphene, *h*-BN, and MoSe_2_, respectively. The top parts of few-layer graphene, *h*-BN and MoSe_2_ including 1, 2, 3, or 4 layers were transversely moved as a whole part with respect to the bottom parts, as shown in Figure 1d, and shifted relatively to different divided positions in the *a*_1_–*a*_2_ planes. Different interlayer stacking were achieved when the top parts shifted to different divided positions. The interlayer distances *d* of the few-layer 2D crystals were modified by changing the *z*-direction coordinates of the atoms in the top layers. For graphene and *h*-BN, 1 C and 1 B atom in the top layers and 1 C and 1 B atom in the bottom layers were fully fixed at each shifted position. For MoSe_2_, only the Se atoms at the top and bottom surfaces were fully fixed. Then those systems were relaxed again by PBE + MBD. After relaxation, the normal forces *F*_n_ at different interlayer distances and shifted positions were calculated by summing the *z*-direction forces of all atoms in the uppermost layers, and the total energies $E_{total}$ of the few-layer 2D crystals at different shifted positions and interlayer distances were calculated. Next, the total energies at a given shifted position were fitted with the corresponding normal pressure *P*_n_ (*P*_n_ *= F*_n_/*A*, *A* is the unit cell area) by a 3-order polynomial, as shown in Figure 2. According to the fitting curves, the total energies at a given normal pressure was obtained. Through this way, the interlayer sliding simulation under constant normal force mode was realized. Then the potential energy surfaces (PES) for the structures with all the shifted positions under constant normal force mode were constructed by $\Delta E=E_{total}-E_{min}$, where $E_{min}$ is the lowest total energy.

## 3. Results and Discussion

**Figure 3:**
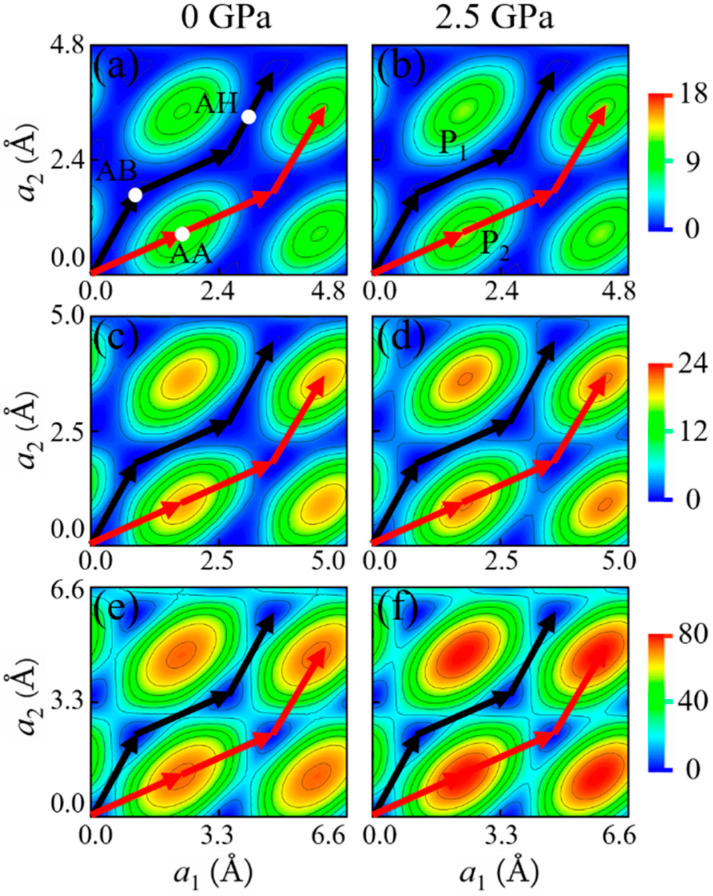
PESs (meV) of 2*L*-layer: (**a**,**b**) graphene, (**c**,**d**) *h*-BN, and (**e**,**f**) MoSe_2_ under the normal pressures of 0 and 2.5 GPa. The black and red lines denote the lowest and highest sliding energy paths, respectively.

**Figure 4:**
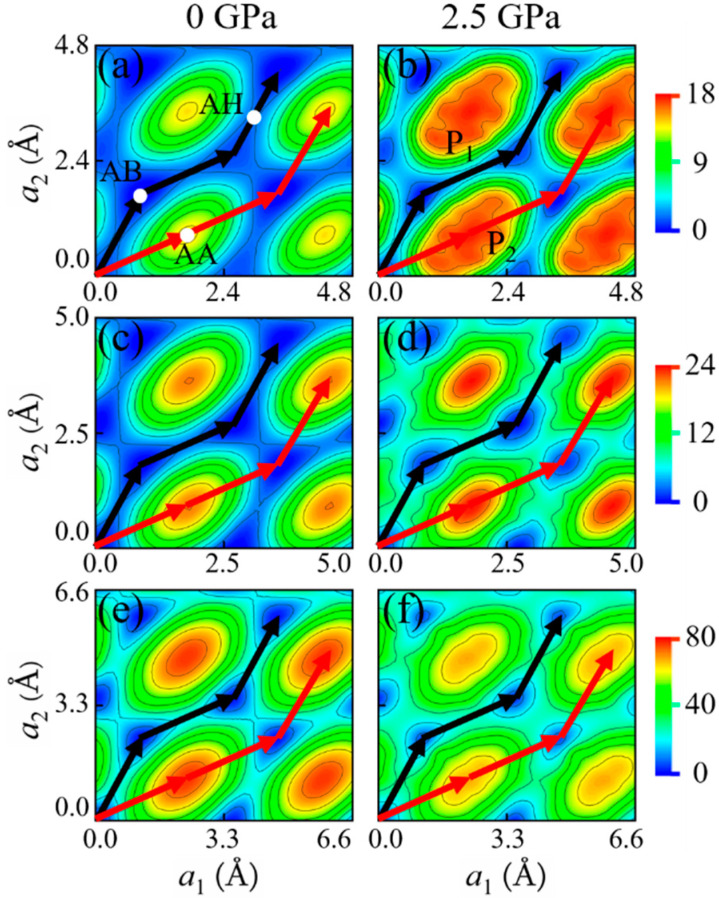
PESs (meV) of 6*L*-layer: (**a**,**b**) graphene, (**c**,**d**) *h*-BN, and (**e**,**f**) MoSe_2_ under the normal pressures of 0 and 2.5 GPa. The black and red lines denote the lowest and highest sliding energy paths, respectively.

**Figure 5:**
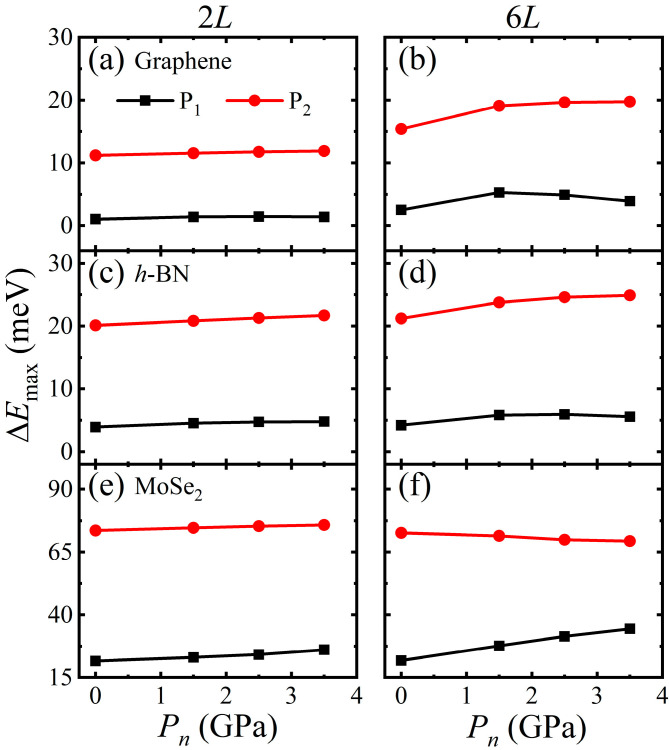
The variations of $\Delta E_{max}^{P_{1}}$ and $\Delta E_{max}^{P_{2}}$ with normal pressure for 2*L*- and 6*L*-layer: (**a,b**) graphene, (**c**,**d**) *h*-BN, and (**e**,**f**) MoSe_2_.

**Figure 6:**
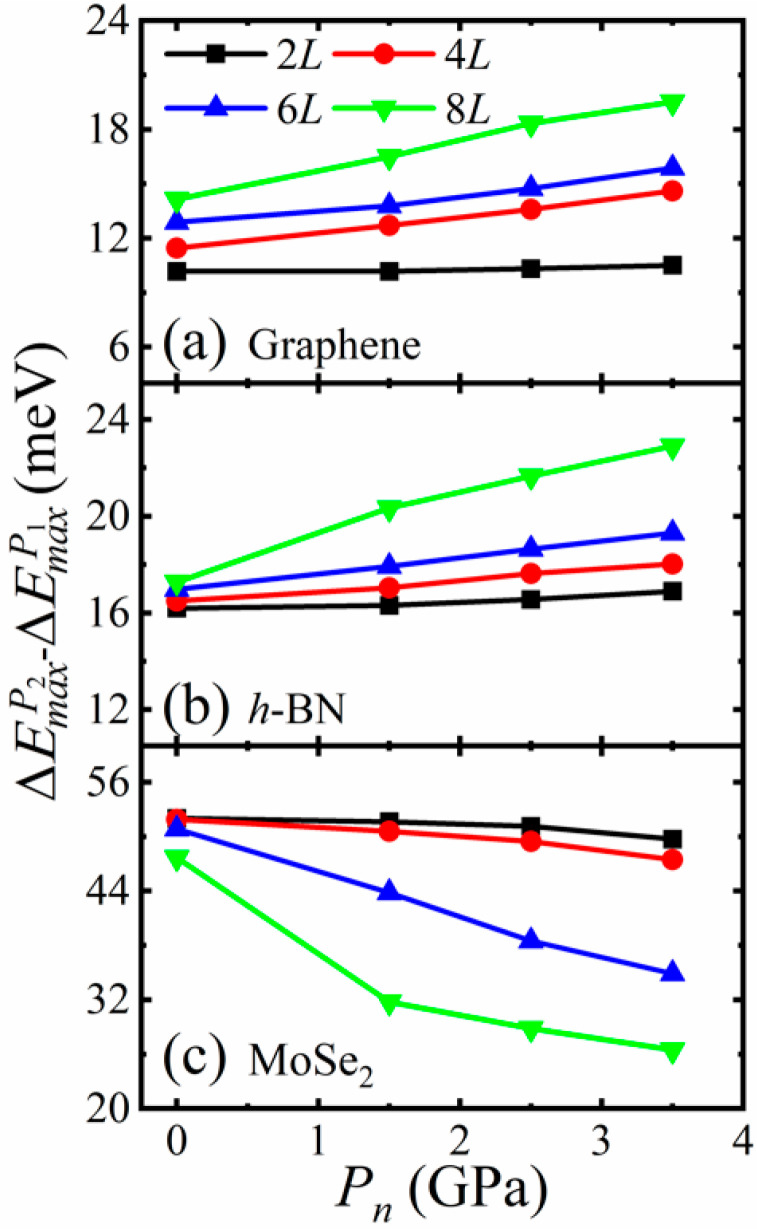
The deviations $\Delta E_{max}^{P_{2}}-\Delta E_{max}^{P_{1}}$ of few-layer: (**a**) graphene, (**b**) *h*-BN, and (**c**) MoSe_2_ with normal pressure.

As shown in Figure 3 and Figure 4, for all of these 2D crystals, the AA stacking has the highest energy while the AB stacking has the lowest energy at a given normal pressure. There are two typical sliding paths *P*_1_ and *P*_2_, see Figure 3 and Figure 4: *P*_1_ is from AB to AB via an apex point AH in which path the energy barrier $\Delta E_{max}^{P_{1}}$ ($\Delta E_{max}^{P_{1}}=E_{total}^{AH}-E_{total}^{AB}$) is the lowest compared with the other paths, another *P*_2_ is AB to AA to AB in which path the energy barrier $\Delta E_{max}^{P_{2}}$ ($\Delta E_{max}^{P_{2}}=E_{total}^{AA}-E_{total}^{AB}$) is the highest. The apex point AH with the highest energy along the sliding path *P*_1_ is defined as AH stacking. Figure 5 shows the variations of $\Delta E_{max}^{P_{1}}$ and $\Delta E_{max}^{P_{2}}$ with normal pressure for 2*L-* and 6*L*-layer graphene, *h*-BN and MoSe_2_. For bilayers, both $\Delta E_{max}^{P_{1}}$ and $\Delta E_{max}^{P_{2}}$ increase with the pressure increases. When the layer number becomes 6, $\Delta E_{max}^{P_{1}}$ of graphene first increases and then decreases with normal pressure. In contrast, $\Delta E_{max}^{P_{2}}$ of MoSe_2_ decreases with the normal pressure increases. The interlayer sliding energy barriers are affected by the change in layer number. On the other hand, the deviation $\Delta E_{max}^{P_{2}}-\Delta E_{max}^{P_{1}}$ between the energy barriers $\Delta E_{max}^{P_{1}}$ and $\Delta E_{max}^{P_{2}}$ is directly related to the interfacial friction anisotropy of the considered few-layer 2D vdW crystals. The higher $\Delta E_{max}^{P_{2}}-\Delta E_{max}^{P_{1}}$ means the stronger friction anisotropy while the lower means the weaker friction anisotropy. It can be seen from Figure 6 that the deviations $\Delta E_{max}^{P_{2}}-\Delta E_{max}^{P_{1}}$ of graphene and *h*-BN increase with the pressure increases, indicating that normal loading enhances the interfacial friction anisotropy. Moreover, $\Delta E_{max}^{P_{2}}-\Delta E_{max}^{P_{1}}$ of graphene and *h*-BN is further increased with the increase of layer number. When the thicknesses of few-layer graphene and *h*-BN becomes thicker, the interfacial friction anisotropy increases as well. On the contrary, the deviation $\Delta E_{max}^{P_{2}}-\Delta E_{max}^{P_{1}}$ of MoSe_2_ decreases with the pressure increases so that normal loading weakens the interfacial friction anisotropy. Meanwhile, the interfacial friction anisotropy of few-layer MoSe_2_ decreases with the increase of layer number. Obviously, the normal pressure and thickness increasing impose completely different influence on the interfacial friction anisotropy of few-layer graphene, *h*-BN, and MoSe_2_.

**Figure 7:**
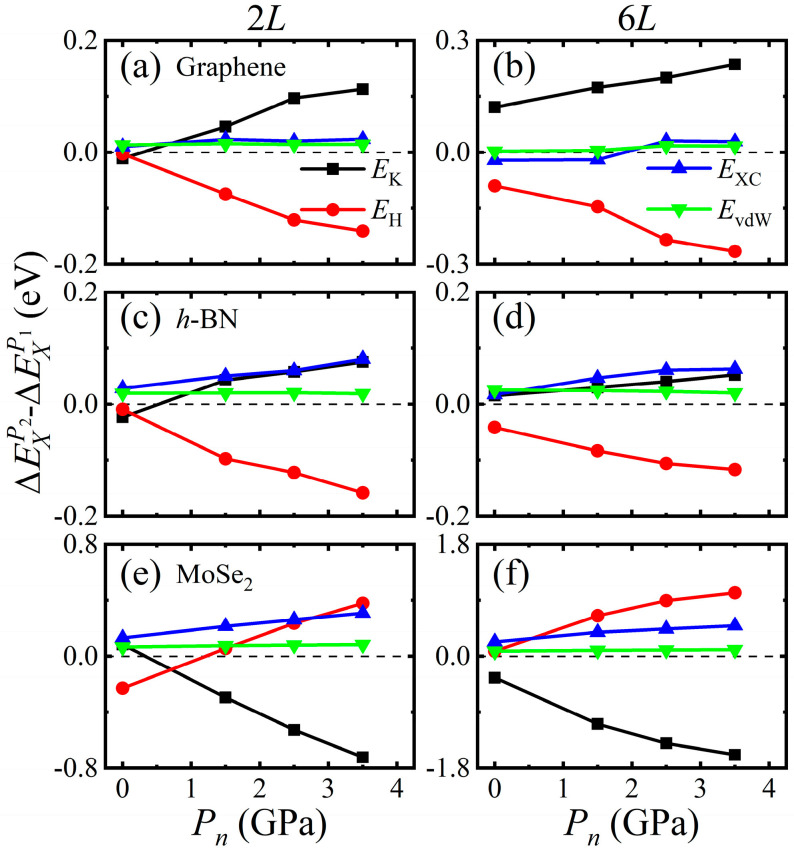
The variations of $\Delta E_{X}^{P_{2}}-\Delta E_{X}^{P_{1}}\;$ with normal pressure for 2*L*- and 6*L*-layer: (**a**,**b**) graphene, (**c**,**d**) *h*-BN, and (**e**,**f**) MoSe_2_.

In our DFT calculations, the total energy $E_{total}$ consists of kinetic, electrostatic, exchange-correlation, and vdW energies, namely $E_{total}=E_{K}+E_{H}+E_{XC}+E_{vdW}$. To better understand the different friction anisotropy in these few-layer 2D crystals, the energy differences in exchange-correlation energy $\Delta E_{XC}$, electron kinetic energy $\Delta E_{K}$, electrostatic energy $\Delta E_{H}$ and vdW energy $\Delta E_{vdW}$ between AH or AA and AB stacking have been calculated by $\Delta E_{X}^{P_{1}}=E_{X}^{AH}-E_{X}^{AB}$ or $\Delta E_{X}^{P_{2}}=E_{X}^{AA}-E_{X}^{AB}$, where *X* = *K*, *H*, *XC*, and *vdW* representing electron kinetic, electrostatic, exchange-correlation, and vdW energies, respectively. Then $\Delta E_{max}^{P_{2}}-\Delta E_{max}^{P_{1}}=\underset{X}{\sum }(\Delta E_{X}^{P_{2}}-\Delta E_{X}^{P_{1}})=\underset{X}{\sum }(E_{X}^{AA}-E_{X}^{AH})$ can be deduced. Figure 7 shows the variations of $\Delta E_{X}^{P_{2}}-\Delta E_{X}^{P_{1}}$ with normal pressure for 2*L-* and 6*L*-layer graphene, *h*-BN and MoSe_2_. For graphene, $\Delta E_{K}^{P_{2}}-\Delta E_{K}^{P_{1}}$ increases but $\Delta E_{H}^{P_{2}}-\Delta E_{H}^{P_{1}}$ decreases as the pressure increases, while $\Delta E_{XC}^{P_{2}}-\Delta E_{XC}^{P_{1}}$ and $\Delta E_{vdW}^{P_{2}}-\Delta E_{vdW}^{P_{1}}$ slightly changes with normal pressure. Similarly, $\Delta E_{K}^{P_{2}}-\Delta E_{K}^{P_{1}}$ and $\Delta E_{XC}^{P_{2}}-\Delta E_{XC}^{P_{1}}$ of *h*-BN increase but $\Delta E_{H}^{P_{2}}-\Delta E_{H}^{P_{1}}$ decreases with the increase of normal pressure. In contrast, $\Delta E_{K}^{P_{2}}-\Delta E_{K}^{P_{1}}$ of MoSe_2_ decreases but $\Delta E_{H}^{P_{2}}-\Delta E_{H}^{P_{1}}$ increases as the pressure increases. Therefore, the opposite contributions and cancelling effects of the electron kinetic energies and electrostatic energies lead to such quite different friction anisotropy between graphene, *h*-BN and MoSe_2_. It is shown from Figure 7 that the changes in pressure and layer number slightly influence $\Delta E_{vdW}^{P_{2}}-\Delta E_{vdW}^{P_{1}}$. The increase in the layer number of the considered 2D crystals modifies the values of $\Delta E_{X}^{P_{2}}-\Delta E_{X}^{P_{1}}$ but will not change the total variation trends.

**Figure 8:**
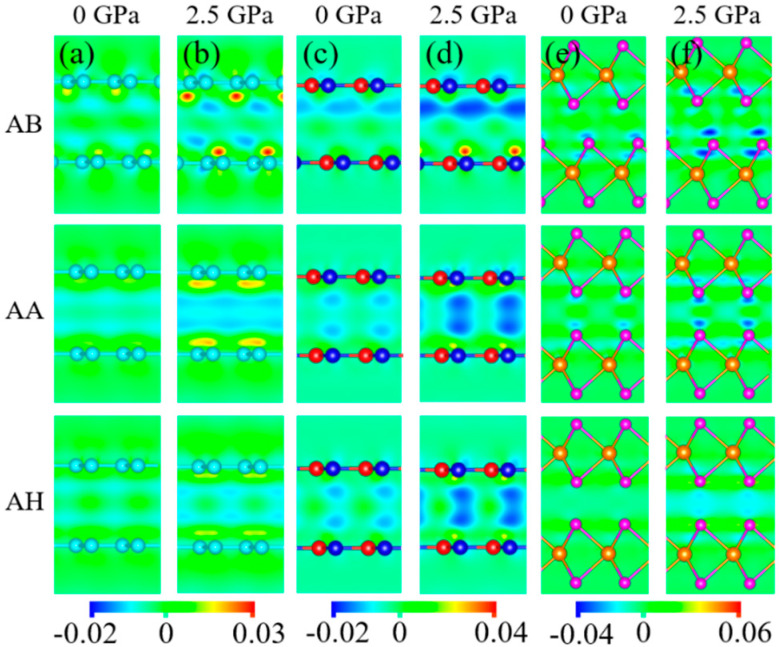
2D projections of interlayer charge density differences (in unit of $e/{Å}^{3}$) for 6*L*-layer: (**a**,**b**) graphene, (**c**,**d**) *h*-BN, and (**e**,**f**) MoSe_2_ with AB, AA, and AH stacking under the normal pressures of 0 and 2.5 GPa.

In order to further elucidate the mechanism of interfacial friction anisotropy, the interlayer charge density differences Δρ for 6*L*-layer graphene, *h*-BN, and MoSe_2_ at AB, AA, and AH stacking have been calculated under the normal pressures of 0 and 2.5 GPa by $\;\Delta \rho =\rho_{6L-layer}-\rho_{top-part}-\rho_{bottom-part}$, where $\rho_{6L-layer}$ is the total charge density, $\rho_{top-part}$ and $\rho_{bottom-part}$ are the charge densities of top three layers and bottom three layers, respectively. As shown in Figure 8, for graphene and *h*-BN the interlayer charge depletions increase with the pressure increases, and more charges move to the interfacial C atoms and N atoms. For MoSe_2_, the interlayer charge accumulations and depletions are relatively weak and charge exchange mainly occurs around the interfacial Se atoms. As a result, the effect of pressure on the interfacial friction anisotropy in few-layer MoSe_2_ is different from that of few-layer graphene and *h*-BN. Moreover, we also considered a 6*L*-layer graphene with initial ABC stacking, *h*-BN with initial AA’ stacking, and MoSe_2_ with initial AA’ stacking. The energy barriers between the interlayer maximum energy sliding paths and minimum energy sliding paths were calculated by the same method and procedure. $\Delta E_{max}^{P_{2}}-\Delta E_{max}^{P_{1}}$ of 6*L*-layer graphene with ABC stacking and *h*-BN with AA’ stacking increases with the normal pressure increases, which is consistent with that of 6*L*-layer graphene and *h*-BN with initial AB stacking. On the contrary, $\Delta E_{max}^{P_{2}}-\Delta E_{max}^{P_{1}}$ of MoSe_2_ with AA’ stacking decreases with the normal pressure increases, which is also consistent with that of 6*L*-layer MoSe_2_ with initial AB stacking. Therefore, the initial stacking mode will not change the qualitative variations of friction anisotropy with normal pressure.

## 4. Conclusions

In summary, we show by comprehensive DFT calculations that under constant normal force mode the interfacial friction anisotropy in few-layer 2D vdW crystals is significantly modified by normal pressure and layer number. For graphene and *h*-BN, the friction anisotropy increases with increasing the normal pressure and layer number. On the contrary, the friction anisotropy for MoSe_2_ decreases with increasing the normal pressure and layer number. The opposite contributions of electron kinetic energies and electrostatic energies to the sliding energy barriers and different interlayer charge exchanges in few-layer graphene, *h*-BN, and MoSe_2_ lead to such remarkable deviation in interfacial friction anisotropy. These results deepen our understanding of friction behaviors of 2D vdW crystals and provide some new insights into the application of 2D vdW crystals as solid lubricants.

## Data Availability

Data is contained within the article.
